# Optical coherence tomography detection of retinal neural loss in patients with tuberous sclerosis

**DOI:** 10.1186/s40942-024-00535-7

**Published:** 2024-02-04

**Authors:** Paula Basso Dias, Anna Carolina Badotti Linhares, Ana Barbara Dias Lopes Urzedo, Rony Carlos Preti, Leandro Cabral Zacharias, Leonardo Provetti Cunha, Mário Luiz Ribeiro Monteiro, Kenzo Hokazono

**Affiliations:** 1 Department of Ophthalmology, Hospital de Clínicas, Federal University of Paraná (HC UFPR), R. da Paz, 195 (123), Curitiba, Paraná 80060-160 Brazil; 2 Department of Ophthalmology, Faculty of Medicine, University of São Paulo (FMUSP), São Paulo, Brazil

**Keywords:** Tuberous sclerosis complex, Retinal nerve fiber layer thickness, Macular thickness, Optical coherence tomography (OCT)

## Abstract

**Purpose:**

Tuberous Sclerosis (TS) is a rare, multisystem genetic disease caused by mutations in the TSC1 and TSC2 genes, leading to abnormalities in cell differentiation and proliferation. This study aimed to evaluate the neural integrity of individuals with TS by using Optical Coherence Tomography (OCT) to examine the peripapillary retinal nerve fiber layer (RNFL) thickness and the macular thickness in patients with TS and to compare with healthy controls.

**Methods:**

Peripapillary and macular OCT scans (Optopol Revo NX SD OCT) were performed on 41 eyes from 22 TS patients, divided into two groups based on the presence of retinal hamartomas, and compared to 20 eyes from a control group. The average peripapillary RNFL thickness was measured for each quadrant. The macular total thickness and ganglion cell layer (GCL) + inner plexiform layer (IPL) thickness were measured based on the Early Treatment Diabetic Retinopathy Study (ETDRS) map. All measurements were then compared between the groups and controls.

**Results:**

The TS group showed significantly reduced RNFL thickness and macular thickness when compared to the control group. Specifically, patients with retinal hamartomas exhibited an even more pronounced thinning of both RNFL and macular thickness.

**Conclusions:**

These findings suggest that TS patients undergo significant changes in retinal neurodevelopment and experience axonal loss. This finding may have significant prognostic utility regarding central nervous system degeneration in TS, particularly among patients with retinal hamartomas. OCT may serve as a valuable tool for assessing axonal structural abnormalities in TS patients.

*Trial Registration Number*: Not applicable.

## Introduction

Tuberous Sclerosis (TS) is a rare multisystem genetic disease characterized by autosomal dominant inheritance caused by mutations in the TSC1 (located on chromosome band 9q34) and TSC2 (located on chromosome band 16p13.3) genes, which leads to changes in cell differentiation and proliferation [1]. These genes encode hamartin and tuberin, respectively. Mutations in these genes lead to the overactivation of the mammalian target of rapamycin (mTOR) signaling pathway, resulting in the subsequent alteration in cell proliferation and differentiation. TS has an estimated incidence of 1 in 5800 to 1 in 20,000 live births. Individuals with TS can develop benign circumscribed lesions in almost all tissues, including the central nervous system (CNS), lungs, skin and heart [1]. Clinically, TSC is characterized by the development of hamartomatous growth in multiple organ systems, including the brain, heart, lung, kidney, skin, and eyes [2].

The ophthalmological manifestations of TS include a range of conditions, such as retinal pigmentation and vascular alterations, optic nerve atrophy, glaucoma, and coloboma of the iris, lens, choroid, and retina [3]. In addition, a classic manifestation of TS is retinal astrocytic hamartoma (RAH), typically presenting as a bilateral and stable condition over time [3]. The clinical presentations of TS are varied, and manifestations continue to develop throughout the affected individuals’ lifetimes. Thus, an accurate diagnosis is essential for appropriate medical surveillance and treatment [2].

The CNS is almost invariably affected in TS. Magnetic resonance imaging (MRI) studies have revealed white matter (WM) changes therein, although these studies do not accurately clarify the extent of damage to the WM [4]. Neuropathological studies in individuals with TS have shown diffuse microstructural abnormalities in the WM, reflecting axonal disorganization, diminished or altered myelination, or gliosis [5, 6]. However, there is still no clear information about whether there are defects in the axons that regulate neuronal networks [4].

The identification of the pathogenic mutation in TSC1 or TSC2 DNA is sufficient to establish a definitive diagnosis of TS, configuring an independent diagnostic criterion. However, in 15–20% of cases, conventional genetic testing does not detect the pathogenic mutation. For such cases, clinical diagnostic criteria have been established as follows: 1. major features: hypomelanotic macules (≥ 3, at least 5 mm in diameter), angiofibromas (≥ 3) or fibrous cephalic plaques, ungual fibromas (≥ 2), shagreen patches, multiple retinal hamartomas, cortical dysplasias, subependymal nodules, subependymal giant cell astrocytoma, cardiac rhabdomyoma, lymphangioleiomyomatosis, and angiomyolipomas (≥ 2) and 2. minor features: “confetti” skin lesions, dental enamel pits (> 3), intraoral fibromas (≥ 2), retinal achromic patches, multiple renal cysts, and nonrenal hamartomas. A definitive diagnosis is made when two major features or one major feature with ≥ 2 minor features are found [2].

Optical coherence tomography (OCT) has emerged as a valuable tool for assessing axonal abnormalities, especially in optic neuropathies. The retinal nerve fiber layer (RNFL) consists of retinal ganglion cell axons that form the optic nerve. As RNFL axons are unmyelinated and directly synapse in the lateral geniculate nucleus, this layer represents an appropriate site for studying the CNS. RNFL thickness values are considered potential structural markers of axonal loss and CNS degeneration in various neurological and neurodevelopmental conditions [7–10]. Moreover, OCT is being used to characterize the features of retinal tumors in vivo, including RAHs.

The purpose of our study was to evaluate RNFL and macular thickness using spectral domain SD-OCT in patients with TS, both with or without retinal hamartomas. To our knowledge, this is the first study to evaluate the macular thickness in patients with TS.

## Methods

This observational, cross-sectional study was approved by our Institutional Review Board Ethics Committee and adhered to the principles of the Declaration of Helsinki. Informed consent was obtained from all participants.

**Fig. 1:**
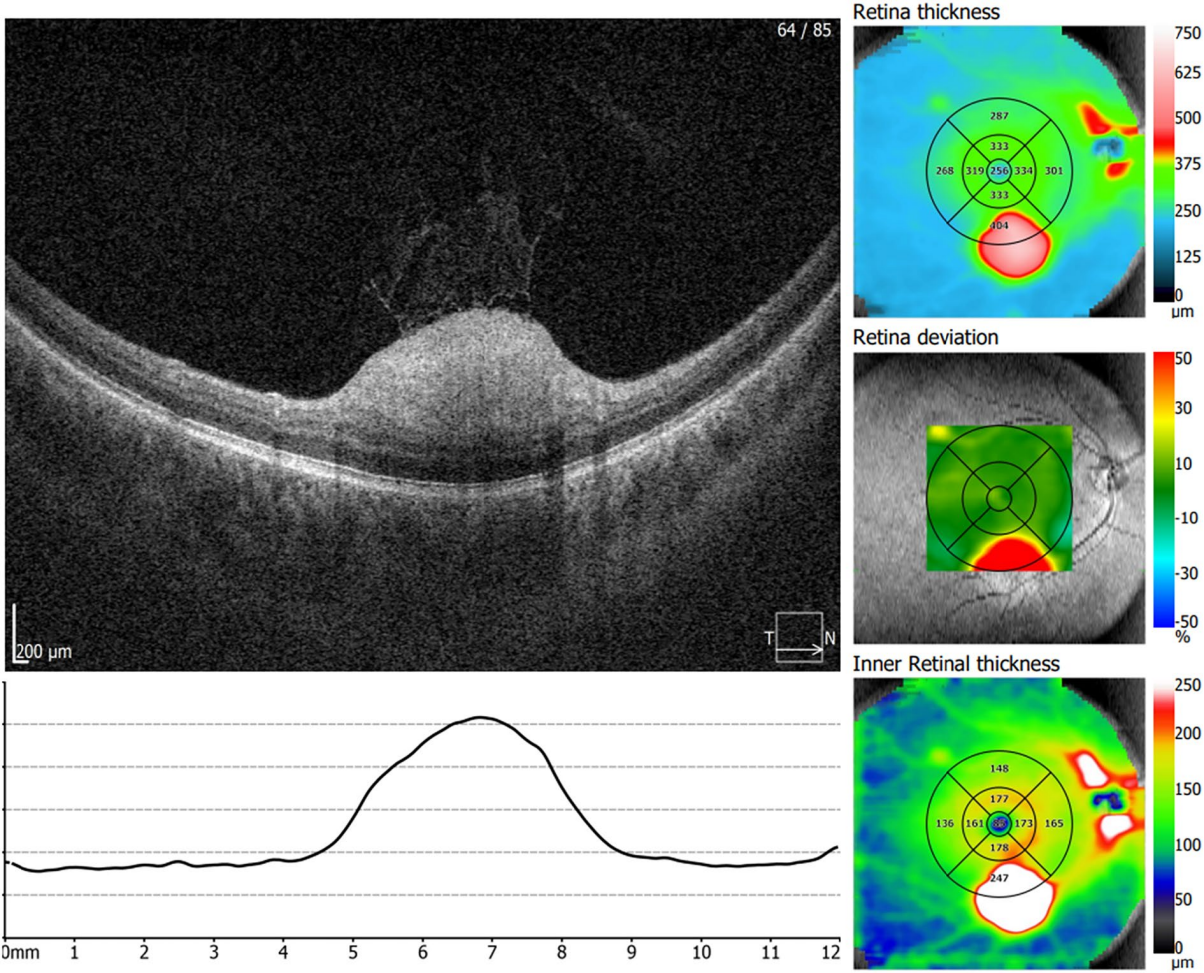
OCT images showing an example of an excluded eye due to macular RAH, which could potentially result in an overestimation of RNFL thickness

A total of 41 eyes from 22 patients with TS and 20 eyes from controls were evaluated. TS was diagnosed based on current diagnostic criteria[2]. Patients with TS were divided into two distinct groups: one with TS but without RAH and the other with TS and RAH, which included patients with any lesion detected during fundoscopy or OCT examination. Within the TS with RAH group, two eyes were excluded from the analysis due to the presence of macular lesions, which could potentially result in an overestimation of RNFL thickness (Fig. 1). Additionally, one patient in the TS group had only one eye studied due to prior evisceration of the contralateral eye after severe ocular trauma. A control group (CG) of twenty individuals without known ophthalmological diseases and with normal OCT examination results was included in the study. The individuals in the CG were paired with the TS group and TS with retinal hamartomas group based on gender, age, and laterality.

**Fig. 2:**
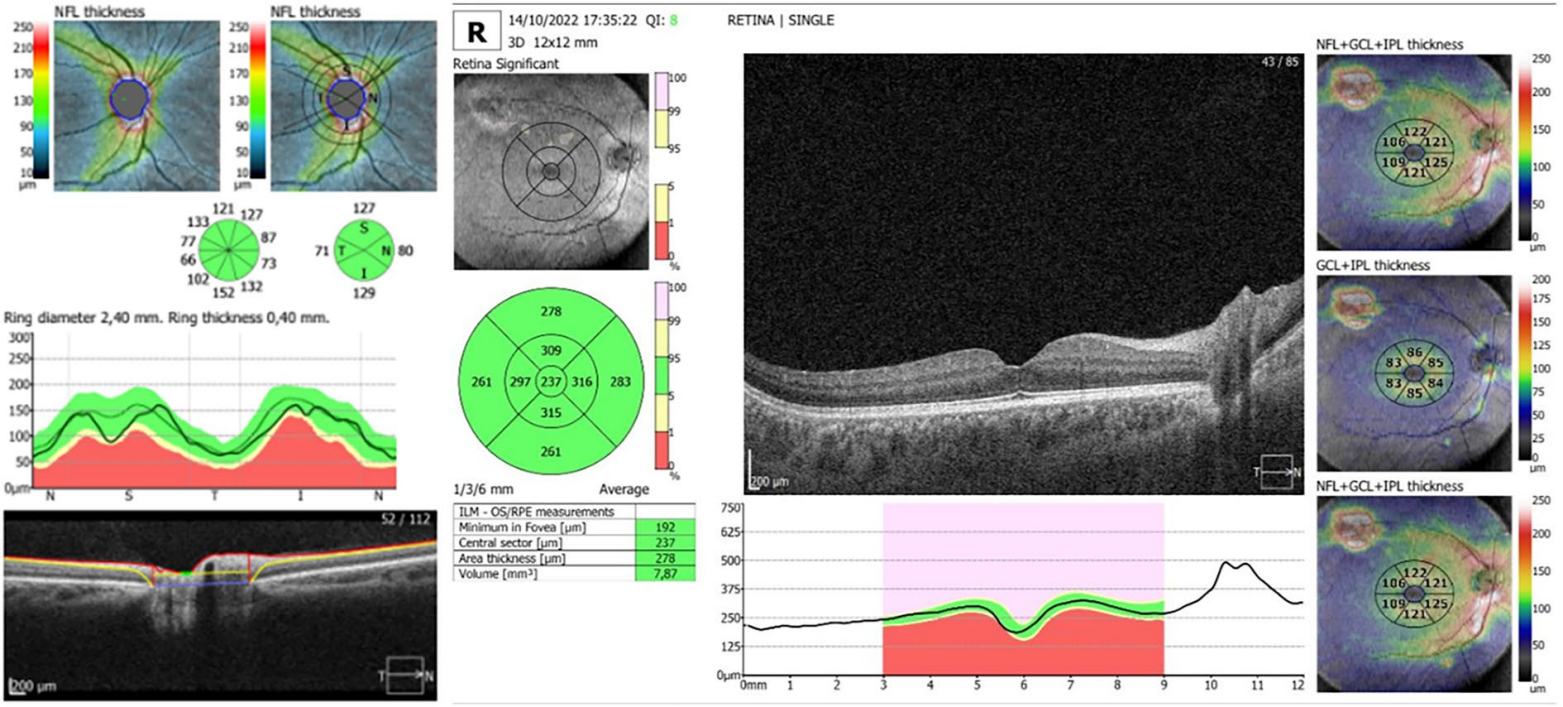
OCT images showing an example of the OCT parameters used to calculate the peripapillary RNFL and macular thickness

Participants underwent high-resolution SD-OCT using the Optopol Revo NX SD-OCT device. For each eye of every participant in the TS group, TS group with RAH, TS group without RAH, and CG, the OCT parameters were automatically calculated by the equipment’s software. RNFL thickness measurements were taken using a circular (Ø = 3.4 mm) peripapillary map when taking measurements corresponding to the overall average thickness (360° measure) and the following Optic Nerve Head (ONH) sectors: temporal (T), superior (S), nasal (N), and inferior (I) (Fig. 2).

Macular thickness measurements were measured based on the Early Treatment Diabetic Retinopathy Study (ETDRS) map. The parameters registered were central, superior inner (SI), inferior inner (II), temporal inner (TI), nasal inner (NI), superior outer (SO), inferior outer (IO), temporal outer (TO), nasal outer (NO), and average macular thickness. The average macular thickness represented the weighted average of the sectoral macular thickness measurements, excluding the fovea. Moreover, the mean retinal ganglion cell layer (GCL) + inner plexiform layer (IPL) was evaluated in patients in the TS group, TS with RAH group, TS without RAH group, and CG (Fig. 2). All scans were conducted by a qualified operator and subsequently reviewed to ensure adequate signal strength as well as proper centering, beam placement, and targeting.

The descriptive analysis included mean values ± standard deviations for normally distributed variables, following verification of the normality assumption with the Shapiro–Wilk test. Statistical analysis was performed with Microsoft Excel 2000 and Graphpad Prism (Graphpad Prism for Windows 5.03). Student’s t-test was used to compare up to two unpaired parametric samples.

## Results

Among the 22 patients included in the TS group, 10 were female (45.4%) and 12 were male (54.6%). Among these patients, 14 (63.6%) presented with retinal hamartomas. The mean age of the participants in the TS group was 23.7 years (± 9.84, range 5.0–46.0 years). The CG comprised 20 patients, with 11 women (55%) and nine men (45%). The mean age of the control group was 23.15 years (± 6.62, range 10.0–32.0 years); see Table 1.

**Table 1:** Table showing clinical characteristics of the TS group, TS with RAH group and CG

	TS Group (n = 41)	TS with RAH GROUP (n = 27)	TS without RAH GROUP (n = 14)	CG (n = 20)	TS group VS CG (p value)	TS with RAH group VS CG (p value)	TS without RAH group VS CG (p value)	TS without RAH VS TS with RAH group (p value)
Clinical characteristics								
Age (years)	23.7 ± 9.84	22.57 ± 9.21	22.13 ± 13.17	23.15 ± 6.62	p = 0.8	p = 0.8	p = 0.8	p = 0.9
Sex (men/women)	12/10	7/7	5/3	9/11				
RNFL values (μm)								
Global	120.7 ± 15.17	120.0 ± 16.75	122.3 ± 11.30	132.4 ± 13.19	p = 0.003*	p = 0.04*	p = 0.08	p = 0.68
Superior quadrant (S)	142.6 ± 18.41	141.6 ± 19.29	144.8 ± 16.87	168.7 ± 19.43	p = 0.003*	p = 0.0007*	p = 0.007*	p = 0.63
Nasal quadrant (N)	99.94 ± 35.55	99.56 ± 42.00	100.8 ± 13.76	104.4 ± 16.70	p = 0.70	p = 0.73	p = 0.60	p = 0.92
Inferior quadrant (I)	147.0 ± 24.11	145.6 ± 26.11	150.2 ± 19.52	173.4 ± 15.52	p = 0.002*	p = 0.003*	p = 0.007*	p = 0.061
Temporal quadrant (T)	78.50 ± 11.84	79.52 ± 13.53	76.8 ± 6.52	82.9 ± 17.85	p = 0.36	p = 0.55	p = 0.26	p = 0.44
Macular values (μm)								
Global macular thickness	291.7 ± 20.0	287.6 ± 22.23	299.5 ± 11.91	306.5 ± 7.56	p = 0.002*	p = 0.0007*	p = 0.05*	p = 0.07
Central quadrant (C)	214.2 ± 23.8	209.6 ± 18.65	223.1 ± 30.30	227.9 ± 21.94	p = 0.04*	p = 0.04*	p = 0.6	p = 0.08
Superior inner quadrant (SI)	297.7 ± 21.5	292.4 ± 23.12	307.8 ± 13.61	314.4 ± 11.04	p = 0.002*	p = 0.0003*	p = 0.13	p = 0.03*
Nasal inner quadrant (NI)	292.4 ± 25.84	288.3 ± 29.04	300.4 ± 16.20	309.8 ± 11.94	p = 0.006*	p = 0.003*	p = 0.06	p = 0.16
Inferior inner quadrant (II)	296.4 ± 24.91	292.9 ± 27.51	303.0 ± 18.01	315.3 ± 10.57	p = 0.002*	p = 0.001*	p = 0.02*	p = 0.22
Temporal inner quadrant (TI)	281.8 ± 23.09	275.9 ± 24.06	293.1 ± 16.49	302.5 ± 11.64	p = 0.0004*	p < 0.0001*	p = 0.06	p = 0.02*
Superior outer quadrant (SO)	313.3 ± 20.20	309.3 ± 21.13	321.1 ± 16.20	323.2 ± 11.59	p = 0.05*	p = 0.01*	p = 0.67	p = 0.07
Nasal outer quadrant (NO)	319.7 ± 22.92	316.6 ± 25.75	325.8 ± 15.20	330.9 ± 9.46	p = 0.04*	p = 0.02*	p = 0.24	p = 0.23
Inferior outer quadrant (IO)	309.5 ± 25.69	308.3 ± 28.35	311.8 ± 20.37	317.5 ± 9.72	p = 0.18	p = 0.17	p = 0.28	p = 0.69
Temporal outer quadrant (TO)	300.3 ± 24.25	295.4 ± 27.53	309.7 ± 12.13	316.9 ± 19.94	p = 0.01*	p = 0.05*	p = 0.2	p = 0.07
Global GCL + IPL	87.97 ± 9.72	87.03 ± 10.50	89.79 ± 8.05	93.76 ± 4.25	p = 0.01*	p = 0.009*	p = 0.07	p = 0.40
Superior GCL + IPL quadrant	88.68 ± 7.49	87.59 ± 7.04	90.79 ± 8.135	94.75 ± 4.99	p = 0.002*	p = 0.0003*	p = 0.09	p = 0.20
Nasal GCL + IPL quadrant	89.83 ± 10.48	88.89 ± 11.53	91.64 ± 8.151	96.08 ± 4.84	p = 0.01*	p = 0.01*	p = 0.06	p = 0.43
Inferior GCL + IPL quadrant	87.55 ± 12.91	88.00 ± 12.98	86.71 ± 13.23	93.00 ± 5.68	p = 0.08	p = 0.12	p = 0.07	p = 0.77
Temporal GCL + IPL quadrant	86.10 ± 10.13	84.61 ± 11.10	88.96 ± 7.47	91.33 ± 5.40	p = 0.04*	p = 0.02*	p = 0.29	p = 0.20

Results presented as mean ± standard deviation (*p < 0,05)

^*^Statistically significant

**Fig. 3:**
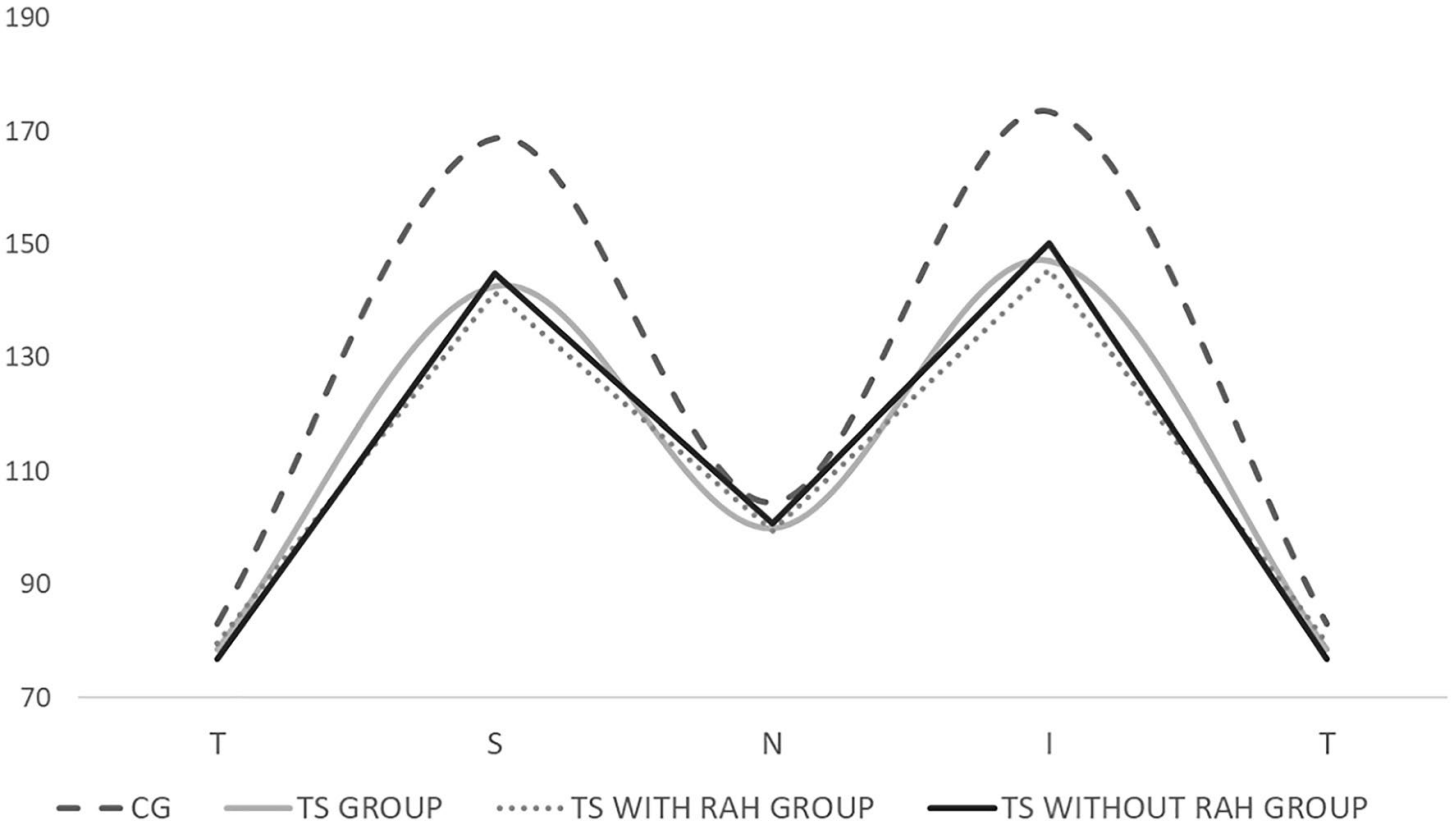
Graph showing mean values of the RNFL thickness in the temporal (T), superior (S), nasal (N), and inferior (I) quadrants in the TS group, TS with RAH group, TS without RAH group, and CG (p < 0.005)

The global average peripapillary RNFL thickness (mean ± standard deviation in micrometers) was significantly lower in the TS group compared to the CG (120.7 ± 15.17 vs. 132.4 ± 13.19; p = 0.003; Table 1). More specifically, when comparing the TS group to the CG, the eyes of TS patients exhibited a significantly thinner peripapillary RNFL in the superior (142.6 ± 18.41 vs. 168.7 ± 19.43; p = 0.003) and inferior (147.0 ± 24.11 vs. 173.4 ± 15.52; p = 0.002) disc quadrants (Table 1 and Fig. 3).

Additionally, the global average peripapillary RNFL thickness (± standard deviation in micrometers) in the TS group with RAH was also significantly lower compared to the CG (120.0 ± 16.75 vs. 132.6 ± 13.19; p = 0.04) (Table 1). Similarly, when comparing the eyes of the TS with RAH group to the CG, there were significantly thinner peripapillary RNFL measurements in the superior (S) (141.6 ± 19.29 vs. 168.7 ± 19.43; p = 0.0007) and inferior (I) (145.6 ± 26.11 vs. 173.4 ± 15.52; p = 0.003) quadrants (Fig. 3).

The global average peripapillary RNFL thickness (mean ± standard deviation in micrometers) in the TS group without RAH was not significantly lower compared to the CG (122.3 ± 11.30 vs. 132.6 ± 13.19; p = 0.08) (Table 1). When comparing the eyes of the TS without RAH group to the CG, there were significantly thinner peripapillary RNFL measurements also only in the superior (S) (144.8 ± 16.87 vs. 168.7 ± 19.43; p = 0.007) and inferior (I) (150.2 ± 19.52 vs. 173.4 ± 15.52; p = 0.007) quadrants (Fig. 3). There was no difference in the RNFL measurements between the TS group with RAH and TS group without RAH.

**Fig. 4:**
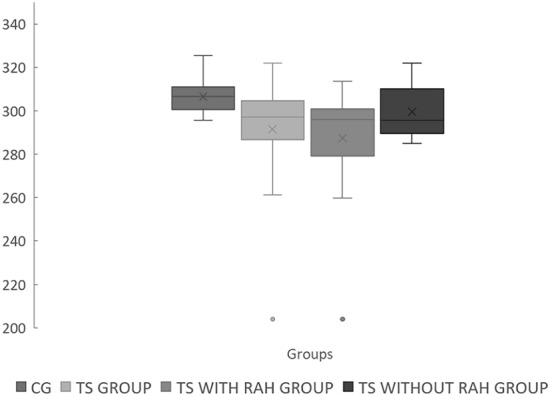
Graph showing mean global macular thickness in the TS group, TS with RAH group, TS without RAH group, and CG (p < 0.05)

The global average macular thickness in the TS group (mean ± standard deviation in micrometers) was significantly lower compared to the CG (291.7 ± 20.00 vs. 306.5 ± 7.57; p = 0.002). Furthermore, when comparing TS with RAH group to the CG, a significantly thinner macular thickness was also observed (287.6 ± 22.23 vs. 306.5 ± 7.57; p = 0.0007) (Fig. 4). When comparing TS without RAH group to the CG, there was also a significantly thinner macular thickness (299.5 ± 11.91 vs. 306.5 ± 7.57; p = 0.005) (Fig. 4). However, when comparing TS with RAH and TS without RAH, there was no difference in macular thickness.

**Fig. 5:**
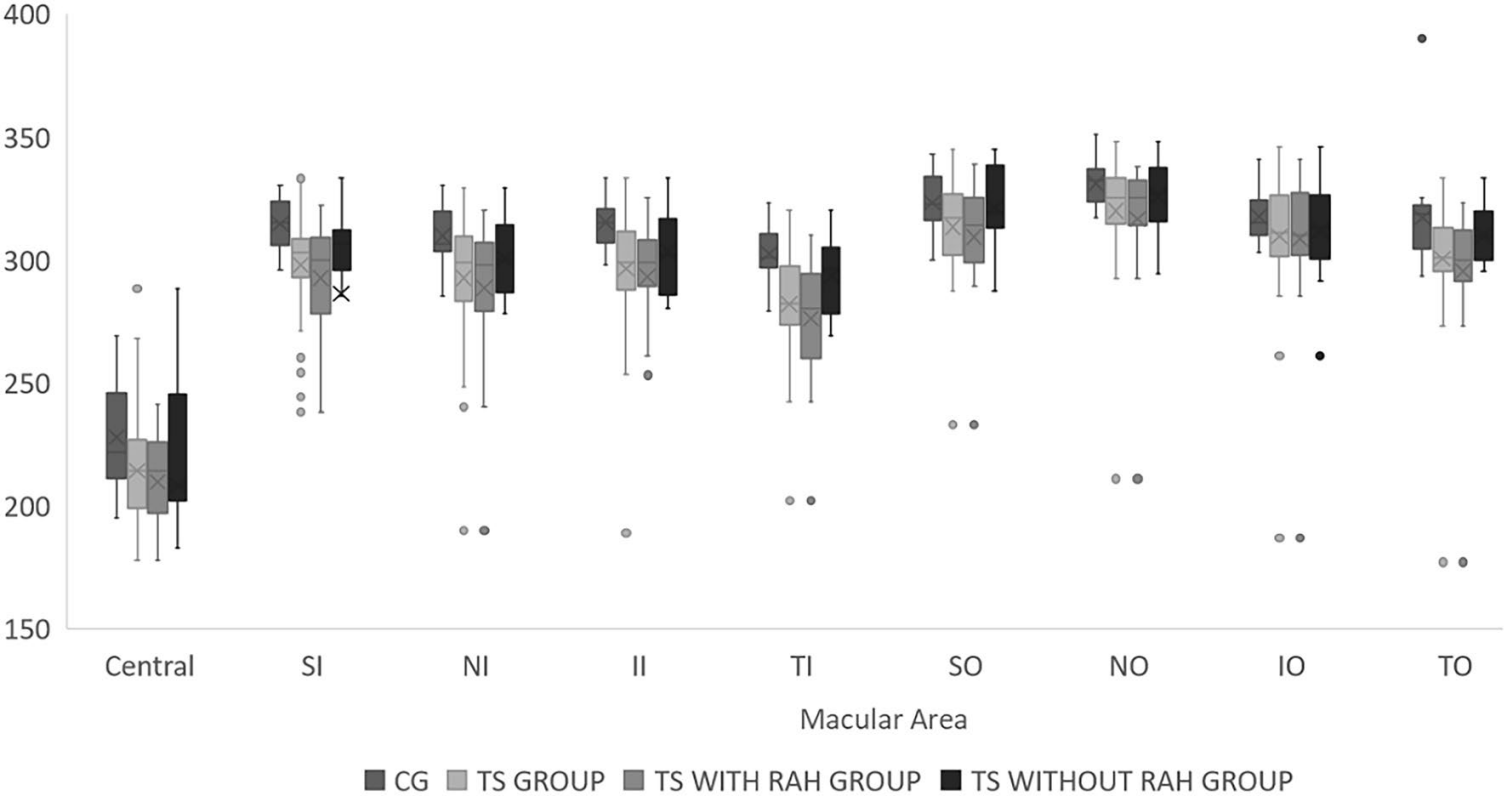
Graph showing mean macular thickness in the central (C), superior inner (SI), nasal inner (NI), inferior inner (II), temporal inner (TI), superior outer (SO), nasal outer (NO), inferior outer (IO), and temporal outer (TO) quadrants in the TS group, TS with RAH group, TS without RAH group, and CG (p < 0.05)

When comparing the TS group to the CG, the eyes of TS patients exhibited significantly macular thinner in the central (C) (214.2 ± 23.8 vs. 227.9 ± 21.94; p = 0.04), superior inner (SI) (297.7 ± 21.5 vs. 314.4 ± 11.04; p = 0.002), nasal inner (NI) (292.4 ± 25.84 vs. 309.8 ± 11.94; p = 0.006), inferior inner (II) (296.4 ± 24.91 vs. 315.3 ± 10.57; p = 0.002), temporal inner (TI) (281.8 ± 23.09 vs. 302.5 ± 11.64; p = 0.0004), superior outer (SO) (313.3 ± 20.20 vs. 323.2 ± 11.59; p = 0.05), nasal outer (NO) (319.7 ± 22.92 vs. 330.9 ± 9.46; p = 0.04) and temporal outer (TO) (300.3 ± 24.25 vs. 316.9 ± 19.94; p = 0.01) quadrants (Fig. 5).

Specifically comparing the TS with RAH group with the CG, it was observed a significantly macular thinner in the central (C) (209.6 ± 18.65 vs. 227.9 ± 21.94; p = 0.04), superior inner (SI) (292.4 ± 23.12 vs. 314.4 ± 11.04; p = 0.0003), nasal inner (NI) (288.3 ± 29.04 vs. 309.8 ± 11.94; p = 0.003), inferior inner (II) (292.9 ± 27.51 vs. 315.3 ± 10.57; p = 0.001), temporal inner (TI) (275.9 ± 24.06 vs. 302.5 ± 11.64; p < 0.0001), superior outer (SO) (309.3 ± 21.13 vs. 323.2 ± 11.59; p = 0.01), nasal outer (316.6 ± 25.75 vs. 330.9 ± 9.46; p = 0.02) and temporal outer (TO) (295.4 ± 27.53 vs. 316.9 ± 19.94; p = 0.05) quadrants (Table 1). However, when comparing TS without RAH with the CG, there was a significantly macular thinner only in the inferior inner (II) (303.0 ± 18.01 vs. 315.3 ± 10.57; p = 0.02) quadrant. When comparing the TS with RAH group to the TS without RAH group, the eyes of TS with RAH patients exhibited significantly macular thinner in the superior inner (SI) (292.4 ± 23.12 vs. 307.8 ± 13.61; p = 0.03) and temporal inner (275.9 ± 24.06 vs. 293.1 ± 16.49; p = 0.02) quadrants (Table 1).

Among the patients in the TS group, the mean thickness of the Ganglion Cell Layer + Inner Plexiform Layer (GCL + IPL) (± standard deviation in micrometers) was significantly lower compared to the CG (87.97 ± 9.72 vs. 93.76 ± 4.25; p = 0.01). Similarly, when comparing TS with RAH group to the CG, a significantly thinner GCL + IPL thickness was observed (87.03 ± 10.50 vs. 93.76 ± 4.25; p = 0.009). However, when comparing TS without RAH group to the CG, there was no difference in GCL + IPL thickness, as well as comparing TS with RAH group and TS without RAH group (Table 1).

Comparing the TS group with the CG, significant thinning of macular GLC + IPL thickness measurements were found in the superior (88.68 ± 7.49 vs. 94.75 ± 4.99; p = 0.002), nasal (89.83 ± 10.48 vs. 96.08 ± 4.84; p = 0.01) and temporal (86.10 ± 10.13 vs. 91.33 ± 5.40; p = 0.04) quadrants. When comparing the TS with RAH group with the CG, significantly thinner GLC + IPL macular measurements were found in the superior (87.59 ± 7.04 vs. 94.75 ± 4.993; p = 0.0003), nasal (88.89 ± 11.53 vs. 96.08 ± 4.84; p = 0.01) and temporal (84.61 ± 11.10 vs. 91.33 ± 5.403; p = 0.02) quadrants. However, no significant difference was found regarding GCL + IPL thickness measurements in any quadrant when comparing TS without RAH group to the CG or in the comparison of TS with RAH group and TS without RAH group (Table 1).

## Discussion

In the present study, we investigated the OCT measured peripapillary RNFL and macular thickness in TS patients with and without RAH. The OCT measurements were significantly lower in TS patients compared to controls. Ocular involvement in TS can manifest in various ways, with RAH being the most common ocular manifestation, occurring in approximately 50% of patients, with 50% of cases showing bilateral retinal involvement [3]. Our study agrees with the literature, as 63.6% of the patients in our sample exhibited RAH. Aronow et al. [11] demonstrated that RAHs are more likely to coexist with subependymal astrocytomas, renal angiomyolipomas, cognitive impairment, and epilepsy, underscoring the importance of accurately identifying RAH and retinal features in TS patients. While several studies have described retinal changes associated with RAH [12–15], the quantification of RNFL and macular thickness has been rarely explored [4].

OCT is a non-invasive, high-quality imaging technique that provides details of the retinal microarchitecture and allows measurement of the RNFL thickness around the disk and macular layer thickness. Several studies based on segmented OCT macular thickness measurements have shown that retinal ganglion cell loss may be an early indicator of neural loss in neurological conditions, such as multiple sclerosis (MS), neuromyelitis optica (NMO) [16], and Alzheimer’s disease [8].

Our results showed RFNL thinning in the superior and inferior disc sectors of subjects with TS compared with healthy controls. Our findings agree with the work of Gialloreti et al. [4], who also found RNFL thinning in TS patients. However, their study did not evaluate the macular measurements. When the macular thicknesses were compared, TS patients presented lower global macular thickness in all sectors evaluated except the IO quadrant. Additionally, our results showed that in patients with RAH, the macular thickness reduction was caused by GCL + IPL thinning, confirming neural damage in TS patients.

Interestingly, TS patients with RAH have demonstrated total retinal thickness and GCL + IPL thickness significant reduced when compared with controls in all macular sectors, while TS patients without RAH did not show a statistically difference in total macular thickness and GCL + IPL thickness when compared with controls, except in the inferior inner quadrant. These findings suggest that TS patients with RAH could be suffering more severe neural loss than TS patients without RAH. Presumably, the presence of hamartomas imposes some degree of stress on the inner retina, causing a process of cellular degeneration and calcification over time, leading to a reduction in the thickness of the inner retinal layers, including the RNFL, which can be detected early by OCT.

Neurological impairments in patients with TS may stem from the presence of tubers, subependymal nodules, and giant astrocytomas, all of which significantly disrupt neuronal function. Interestingly, even regions of WM that appear normal upon inspection have exhibited subtle damage when examined with diffusion techniques in MRI. These findings suggest a diffuse pattern of microstructural changes, including axonal irregularities and loss, as well as altered myelination or gliosis. Such widespread microstructural changes may contribute to the neurocognitive deficits observed in some patients with TS [17, 18] Although MRI diffusion is an objective test, it is expensive, time-consuming, and requires experienced examiners to interpret the images. Our findings suggest that retinal OCT measurements can be a non-invasive biomarker of neural damage in TS patients. However, as a cross-sectional study, our results do not allow us to establish a clear relationship between retinal damage and neurophysiological outcomes, either ocular or systemic. Consequently, it is not possible to assert that these changes correspond directly with the severity of the disease. The only notable correlation pertains to the presence or absence of RAH.

The main cause of neural deficits in TS patients remains incompletely understood; however, a lack of appropriate neuronal development and/or chronic axonal degeneration can be confirmed by our results and other studies using MRI. Longitudinal studies are needed to determine whether axonal damage is a continuous, long-term process in TS patients.

Our study has strong points as a complete evaluation of macular thickness beyond the RNFL around the optic nerve and a relatively high number of TS patients. However, this was an observational, cross-sectional study that did not correlate the detected neural loss in TS patients with systemic neurological deficits, such as motor or cognitive functional defects. Additionally, the cross-sectional design of the study does not permit tracking the modifications in OCT measurements over time.

## Conclusion

Our study included a substantial number of TS patients, which enabled us to uncover new insights. We observed a significant reduction in the RNFL thickness around the optic disk as well as total retinal macular thickness and GCL + IPL thickness in TS patients. Furthermore, the magnitude of this difference was even more significant when comparing the CG to TS patients with retinal hamartomas. By analyzing these alterations in retinal structures, including the RNFL thickness and macular thickness, we can potentially gain valuable information regarding the severity of the disease in individual patients. Additionally, the RNFL thickness around the optic nerve and macular thickness could be potential structural markers for axonal loss in TS patients.

## Data Availability

The data substantiating this study have been incorporated within the article itself, and can be located in references 1 through 18.

## Abbreviations

C: Central
CG: Control group
CNS: Central nervous system
ETDRS: Early Treatment Diabetic Retinopathy Study
GLC: Ganglion cell layer
I: Inferior
II: Inferior inner
IO: Inferior outer
IPL: Inner plexiform layer
MRI: Magnetic resonance imaging
MS: Multiple sclerosis
N: Nasal
NI: Nasal inner
NO: Nasal outer
NMO: Neuromyelitis optica
OCT: Optical coherence tomography
ONH: Optic nerve head
RAH: Retinal astrocytic hamartoma
RNFL: Retinal nerve fiber layer
S: Superior
SI: Superior inner
SO: Superior outer
T: Temporal
TI: Temporal inner
TO: Temporal outer
TS: Tuberous sclerosis
TSC: Tuberous sclerosis complex
WM: White matter
